# Effects of Ramadan fasting on mental well-being in patients with diabetes

**DOI:** 10.1192/j.eurpsy.2026.11070

**Published:** 2026-07-31

**Authors:** F. Zaouali, I. Anes

**Affiliations:** 1 Emergency department and mobile emergency and resuscitation service, Tahar Sfar University Hospital, Mahdia; 2 Outpatient psychiatric consultation, Hadj Ali Soua Regional Hospital, Ksar Hellal, Monastir, Tunisia

## Abstract

**Introduction:**

Ramadan fasting (RF) is widely practiced among Muslim patients with diabetes, yet its effects on mental well-being are not well defined. Evidence from healthy populations suggests variable outcomes in mood, stress, and fatigue. Understanding these effects in diabetic patients is crucial for safe and effective care.

**Objectives:**

To systematically review the impact of Ramadan fasting on depression, anxiety, stress, and diabetes-related distress in patients with diabetes.

**Methods:**

We systematically searched PubMed, Scopus, Web of Science, and PsycINFO up to December 2024 using “Ramadan fasting” AND “diabetes” OR “type 1 diabetes” OR “type 2 diabetes” AND “mental health” OR “psychological well-being” OR “anxiety” OR “depression” OR “stress.” Included studies involved adult diabetic patients observing Ramadan with reported mental health outcomes; reviews, editorials, case reports, and studies on non-diabetic populations were excluded. Data extracted included study design, sample size, demographics, type of diabetes, assessment tools (PHQ-9, PAID-20, DASS-21), and outcomes. Quality was assessed using the Newcastle-Ottawa Scale and Cochrane risk-of-bias tool.

**Results:**

Seven studies (2,148 patients) met inclusion criteria. Five reported improvements in mental well-being, while two showed neutral or negative effects in patients with poor glycemic control or pre-existing psychological conditions. In Pakistan, Yousuf et al. observed depression decreased from 45% to 23%, anxiety from 45% to 26%, and stress from 49% to 35% post-Ramadan among fasting patients; non-fasting controls had no significant changes. In Kuwait, Al-Ozairi et al. reported significant reductions in depression and diabetes-related distress. In Bangladesh, Sultana et al. found 34.5% had diabetes-related distress and 28.2% depressive symptoms, linked to poor glycemic control, complications, elevated stress, and financial difficulties. Improvements were often attributed to spiritual, social, and emotional factors. Findings were heterogeneous, highlighting individual and contextual variability.

**Conclusions:**

Ramadan fasting is generally safe and may improve mental well-being in patients with diabetes, particularly by reducing depression, anxiety, and diabetes-related distress. Adverse effects may occur in those with pre-existing psychological issues or poor glycemic control, emphasizing the need for pre-Ramadan assessment, education, and individualized guidance. Future randomized trials are warranted to confirm causality and assess long-term outcomes.

**Disclosure of Interest:**

None Declared

